# Ankle fracture surgery performed by orthopaedic residents without supervision has comparable outcomes to surgery performed by fellowship trained orthopaedic surgeons

**DOI:** 10.1007/s00402-024-05259-9

**Published:** 2024-05-04

**Authors:** Shay A. Tenenbaum, Yorye Shenkar, Itay Fogel, Or Maoz, Snir Balziano, Yuval Barzilai, Dan Prat

**Affiliations:** Department of orthopedic surgery, Chaim Sheba medical center at Tel Hashomer, Tel Aviv University Faculty of medicine, Ramat Gan, 5262100 Tel Aviv, Israel

**Keywords:** Ankle fractures, Resident training, Competency, Ankle fracture surgery, Autonomy, Complications

## Abstract

**Background:**

Unstable fractures often necessitate open reduction and internal fixation (ORIF), which generally yield favourable outcomes. However, the impact of surgical trainee autonomy on healthcare quality in these procedures remains uncertain. We hypothesized that surgery performed solely by residents, without supervision or participation of an attending surgeon, can provide similar outcomes to surgery performed by trauma or foot and ankle fellowship-trained orthopaedic surgeons.

**Methods:**

A single-center cohort of an academic level-1 trauma center was retrospectively reviewed for all ankle ORIF between 2015 and 2019. Data were compared between surgery performed solely by post-graduate-year 4 to 6 residents, and surgery performed by trauma or foot and ankle fellowship-trained surgeons. Demographics, surgical parameters, preoperative and postoperative radiographs, and primary (mortality, complications, and revision surgery) and secondary outcome variables were collected and analyzed. Univariate analysis was performed to evaluate outcomes.

**Results:**

A total of 460 ankle fractures were included in the study. Nonoperative cases and cases operated by senior orthopaedic surgeons who are not trauma or foot and ankle fellowship-trained orthopaedic surgeons were excluded. The average follow-up time was 58.4 months (SD ± 12.5). Univariate analysis of outcomes demonstrated no significant difference between residents and attendings in complications and reoperations rate (*p* = 0.690, *p* = 0.388). Sub-analysis by fracture pattern (Lauge-Hansen classification) and the number of malleoli involved and fixated demonstrated similar outcomes. surgery time was significantly longer in the resident group (*p* < 0.001).

**Conclusion:**

The current study demonstrates that ankle fracture surgery can be performed by trained orthopaedic surgery residents, with similar results and complication rates as surgery performed by fellowship-trained attendings. These findings provide valuable insights into surgical autonomy in residency and its role in modern clinical training and surgical education.

**Level of evidence:**

Level III – retrospective cohort study.

**Supplementary Information:**

The online version contains supplementary material available at 10.1007/s00402-024-05259-9.

## Introduction

Ankle fractures are among the most common injuries treated by orthopaedic surgeons, with the ankle joint being the most commonly injured weight-bearing joint [3]. Open reduction and internal fixation (ORIF) are typically performed when surgery is indicated. While good clinical outcomes have been extensively reported in the literature [10, 19, 21], minor and major complications (i.e. thromboembolic events, infection, incomplete wound healing, fracture malreduction, malunion, non-union, nerve injury, etc.) can occur.

As Ankle fractures are so common, training orthopaedic surgery residents to successfully treat these fractures once they graduate to become independent caregivers is mandatory. The Accreditation Council for Graduate Medical Education determined that a minimum of 15 cases as “first assist” is required for residency graduation [12]. This is yet another example of resident training based on an apprenticeship model. However, one must not ignore the inherent conflict that exists between surgical training and optimal treatment outcomes with maximal patient safety.

The concern of whether residents’ involvement in surgery increases complication rates and adversely affects outcomes is a subject of research in recent years.

Louie et al. [12]. studied ankle fractures treated with open reduction and internal fixation. The authors showed that surgery time was longer in the resident group, but the overall complication rate was lower. Similarly, Edelstein et al. [5]. showed that resident involvement in orthopaedic surgery decreases the risk of perioperative complications and mortality. Also, other authors reported similar results [7, 13].

Conversely, other published studies [1, 9, 14–18] showed that resident involvement in surgery adversely affects outcomes and complication rates.

However, all the above-mentioned studies share a fundamental methodological flaw. While the present study compares the outcome of surgically treated ankle fractures performed solely by residents to surgeries performed by specialists, previous studies evaluate the effects of residents’ participation in surgery, rather than residents’ performance alone. Therefore, any conclusions drawn from these studies regarding residents’ effect on surgical outcomes, should be considered with caution. The present study hypothesized that surgeries performed solely by residents did not have an increased rate of complications or inferior results to surgeries performed by specialists. The present study’s findings will help improve the understanding of surgical autonomy in residency, and its role in training and education.

## Materials and methods

### Study cohort

From August 1st, 2015, to June 31st, 2019, data on patients with surgically treated ankle fractures were collected and retrospectively reviewed after Institutional review board approval was obtained.

Patients were identified using the International Classification of Diseases, 9th Revision, Clinical Model (ICD-9-CM) coding for ‘fracture of ankle’ (824.x). The diagnosis was confirmed based on the patient’s medical chart and preoperative radiographs. Pediatric cases, revisions, and pilon fracture cases were excluded. Fractures were classified according to the Lauge-Hansen classification [11]. Current Procedural Terminology (CPT) codes were utilized to identify the surgical treatment. The surgical treatment was confirmed with a review of the patient’s chart and postoperative radiographs. All radiographs were evaluated twice, by an orthopaedic resident and a fellowship-trained foot-and-ankle surgeon, and interobserver correlation was calculated.

We categorized cases based on the composition of the surgical team, specifically identifying the participating surgeon as either an orthopaedic resident or a fellowship-trained orthopaedic surgeon specializing in trauma or foot and ankle surgery. Residents were further stratified based on their Postgraduate Year (PGY) level.

Other categorical variables included gender, the American Society of Anesthesiologists Physical Status Classification System (ASA), comorbidities such as osteopenia and osteoporosis, diabetes, and tobacco use history. Continuous variables included patient age and body mass index (BMI), time to surgery from admission to the emergency department, and length of stay.

We collected intraoperative parameters such as tourniquet time and method of fixation. In all surgeries performed by residents, operative notes were specifically analyzed to mention if an “on-call” attending surgeon was called to assist during surgery.

The primary outcomes of this study were mortality, complications (major and minor), and revision surgery. Mortality was confirmed using a national database. Major Complications included: Deep infection, venous thromboembolic event (VTE), myocardial infarction (MI), cerebrovascular accident (CVA), Arrhythmia (i.e., Atrial flutter, atrial fibrillation), pneumonia and congestive heart failure (CHF). Minor complications included: superficial wound infection, delirium, urinary tract infection (UTI), ileus, and bedsores.

We evaluated the reduction of the malleoli and the syndesmosis in plain radiographs. We used the routine follow-up radiographs in standard anterior-posterior, mortise, and lateral views. Reduction quality was categorized as (1) anatomic reduction; (2) gap, step-off, or shortening of less than 2 mm; (3) gap, step-off, or shortening of more than 2 mm.

Primary and secondary outcomes for this study were analyzed for the entire cohort and each subgroup by fracture type, and the number of malleoli fractured and fixed (e.g., Lauge-Hansen supination-external-rotation [SER] isolated lateral malleolus fractures, bimalleolar fractures, and trimalleolar fractures).

### Residency program and healthcare structure

The research was carried out at a tertiary trauma center catering to a population exceeding 2 million individuals. Affiliated with a prominent university, the medical center hosts numerous teaching and academic sessions integral to an accredited orthopaedic surgery residency program. Our institute handles approximately 120 ankle fracture cases annually, predominantly treated during after-hours operating room sessions. These sessions are supervised by senior surgeons three times a week and by residents four times a week, with junior residents acting as first assistants for both senior-level surgeons and senior residents during procedures.

At our institution, the surgical training for residents spans supervised teaching during the initial three post-graduate years (out of a 6-year program). Upon reaching PGY-4, residents, under the program director’s approval, commence independent surgical procedures without direct supervision from senior surgeons, where the senior surgeon may not be present at the hospital during the surgery.

To assess the preparedness of PGY-4 residents for independent surgical practice, a comprehensive evaluation process was established. The program director adopted a multifaceted strategy, incorporating diverse metrics and assessments. Residents had to pass three national tests similar to the OITE (one per year of residency) to gauge their theoretical knowledge and grasp of orthopedic fundamentals, ensuring a robust foundational competence. Alongside these tests, senior attending surgeons consistently evaluated residents’ performance in the operating room over the initial three years, considering factors like surgical proficiency, decision-making skills, and patient care. Additionally, by the culmination of PGY-3, residents typically conducted an average of 10 to 15 “qualifying” surgeries under direct supervision and guidance, tailored to individual performance levels. Following feedback from supervising surgeons, the program director grants permission accordingly. This comprehensive process assessed PGY-4 residents’ readiness for independent surgical practice, synthesizing theoretical expertise, clinical proficiency, surgical skills, and adept decision-making in diverse clinical scenarios. Once residents are approved to perform surgery independently, they remain supervised by another (more senior) resident, for 6 months.

In contrast to several other residency programs, both locally and internationally, where resident autonomy in surgeries of low to mid-level complexity tends to be limited, our program was carefully developed with a balanced approach in mind. This unique strategy aims to provide residents with essential surgical exposure while maintaining a strong emphasis on patient safety measures. We believe that our approach, although still evolving, shows promising outcomes within our specific institutional context.

It’s essential to underscore that a board-certified orthopaedic surgeon is available ‘on call’ round-the-clock, capable of reaching the operating room within 30 min. Prior to each case, a detailed surgical plan is deliberated with the attending surgeon, and informed consent is obtained. Patients and their families are informed that the primary surgeon may be a resident, duly trained to conduct the surgery independently under indirect specialist supervision. Furthermore, all cases undergo formal post-procedure evaluation and feedback by attending surgeons.

As the study conducted in Israel, the healthcare system operates under a universal healthcare framework, where participation in a medical insurance plan is compulsory for all residents. This comprehensive system ensures that all citizens have access to basic healthcare as a fundamental right. Patients treated at the academic hospital included in our study are covered under one of the country’s four official health insurance organizations. These organizations operate as not-for-profit entities, catering to insured individuals across a spectrum of insurance categories, including private payors, state-sponsored insurance plans, and coverage for the uninsured. The mandatory nature of the healthcare insurance system ensures that individuals, including those receiving care in our academic setting, have access to necessary medical interventions and treatments irrespective of their insurance status. Therefore, insurance and coverage status remained consistent across both the fellowship-trained surgeon and senior resident groups and insurance charges did not influence treatment choices whatsoever.

### Statistical methods

Univariate analysis of the surgical team was performed using independent sample T-tests or analysis of variances for all continuous variables: Pearson chi-square was used for categorical variables in each of the surgical subgroups. A p-value of < 0.05 was considered significant. Subsequently, mortality was analyzed using a Kaplan-Meier survival analysis for each subgroup. Log-rank tests were performed to test differences in Kaplan-Meier estimates between groups.

Statistical analysis was performed using the SPSS software package, version 26.0 (SPSS, IBM. Armonk, NY, USA).

## Results

### Patient demographics

Between August 1st, 2015, to June 31st, 2019, 479 ankle fractures met the inclusion criteria. Nineteen cases (4.0%) were operated on by senior orthopaedic surgeons who are not trauma or foot and ankle fellowship-trained orthopaedic surgeons and therefore excluded. Within the remaining 460 cases, residents operated without supervision in 152 cases (33.0%), and 308 cases (67.0%) were operated by fellowship-trained surgeons: 48 cases (10.4%) by foot ankle specialists, and 260 cases (56.5%) by orthopedic trauma surgeons. The time to surgery was 2.25 days (median 1.85, SD ± 2.29) and similar between the groups (*p* = 0.084).

Residents operated more often in the after-hours (after 3 pm) and at night (65.1% and 14.5% of the cases) compared to the specialists (60.1% and 0.6% of the cases), *p* < 0.001. The average follow-up time was 58.4 months (median 59.0, SD ± 12.5) and was similar between the groups.

While ASA scores 3–4 were more prevalent in the resident group (24.6% of the cases vs. 14.2% in the specialist group, *p* = 0.014), other demographic parameters were similar between the study groups (Table 1a, Table 1b).

**Table 1a:** Demographics and Time to Surgery * *p* < 0.05

Group	Specialists N = 308		Residents N = 152		
	Mean	±SD	Mean	±SD	*p*-value
Age	50.2	16.4	50.6	17.7	0.808
BMI	27.4	4.7	27.6	4.6	0.705
Time to Surgery (Days)	2.37	2.40	2.00	2.05	0.084

Abbreviations: BMI – Body Mass Index

**Table 1b:** Demographics and Baseline Functional Status * *p* < 0.05

Group	Specialist *N* = 308		Residents *N* = 152		
	**N**	**%**	**N**	**%**	***p*** **-value**
Gender					
Female	161	52.3%	88	57.9%	0.255
ASA					
1–2	212	85.8%	89	75.4%	0.014*
3–4	35	14.2%	29	24.6%	
Diabetes Mellitus	35	11.4%	16	10.5%	0.788
History of Tobacco Use	40	13.0%	11	7.2%	0.065
Osteopenia or Osteoporosis	9	2.9%	9	5.9%	0.130

Abbreviations: ASA – American Society of Anesthesiologists Physical Status Classification System

In total, in 152 cases, residents were lead surgeons without specialist supervision. Of the cases operated by residents alone, 95 cases (62.5%) were operated by the lead of a PGY 6, 38 (25.0%) by a PGY 5, 17 (11.2%) by a PGY 4, and two cases (1.3%) by a PGY 3.

#### Fracture types

There were similar rates of open fractures in the resident group compared to the specialist group (*p* = 0.354). The distribution of fracture patterns was similar between the groups (*p* = 0.993). The number of fractured malleoli was also similar (*p* = 0.529). (Table 2)

**Table 2:** Fracture Types * *p* < 0.05

Group	Specialist *N* = 308		Residents *N* = 152		
	**N**	**%**	**N**	**%**	***p*** **-value**
Open Fractures	9	2.9%	7	4.6%	0.354
Lauge Hansen^13^					
SER	229	75.8%	114	76.5%	0.993
SAD	16	5.3%	8	5.4%	
PER	43	14.2%	21	14.1%	
PAB	14	4.6%	6	4.0%	
Number of Fractured Malleoli					0.529
Uni-malleolar	127	41.2%	62	40.8%	
Bimalleolar	154	50.0%	81	53.3%	
Trimalleolar	27	8.8%	9	5.9%	

Abbreviations: SER – Supination External Rotation; SAD – Supination Adduction; PER – Pronation External Rotation; PAB – Pronation Abduction

### Intraoperative data

#### Fixation technique

The fixation technique used for the medial malleolus fractures was similar between the groups. Overall, 2 cannulated screws were used in 72.8% of the cases, a tension band wire fixation technique in 16.2%, and an anti-glide plate in 5.9%.

For the lateral malleolus, anatomic lateral malleolus plates (i.e. stainless still Arthrex anatomic locking plates) were used in most cases (89.7%), the specialists used interfragmentary screws more often (68.4%) compared to the residents (45.2%, *p* < 0.001). Within the lateral malleolus fixation, the residents used 1/3 tubular plates less frequently compared to the specialists (2.4% vs. 7.9%, *p* = 0.032).

The posterior malleoli were fixed similarly in both groups (anatomic plate in 43.1%, 1/3 tubular in 51.7%, and a screw in 5.2% of the cases, *p* = 0.140). The use of a syndesmotic fixation device was similar in both groups (*p* = 0.466). The use of a single screw was the most common overall in both groups (74.8%), followed by a suture button device (9.0%) and 2 screws (7.7%). There were merely two instances of conversions from external fixation to ORIF, with one occurrence observed in each group.

##### Reduction quality

Patients’ cases underwent assessment and were categorized into three groups: those achieving anatomic reduction, instances of malreduction within 2 mm, and cases exhibiting malreduction greater than 2 mm. There were 7 cases (3.6%) in the specialist group and 5 cases (5.1%) in the resident group (*p* = 0.556) in which the malreduction of the medial malleolus was within 2 mm of an anatomic reduction. There were no cases with a greater than 2 mm malreduction of the medial malleolus. In one case (0.4%) in the specialist group, there was a malreduction of more than 2 mm of the lateral malleolus, versus 0 cases in the resident group. We identified 7 cases (2.5%) of malreduction within 2 mm in the specialist group versus 7 cases (5.1%) in the resident group (*p* = 0.298). We did not find cases of malreduction of the posterior malleolus in both groups. Regarding the reduction of the syndesmosis, we found 2 cases (3.2%) in the resident group with a malreduction within 2 mm malreduction and one case (1.6%) of malreduction of more than 2 mm. We also identified 2 cases (1.8%) in the specialist group, with malreduction of less than 2 mm (*p* = 0.323) (Table 3).

**Table 3:** Malreduction rates

Group	Specialist N = 308		Residents N = 152		
	**N**	**%**	**N**	**%**	***p*** **-value**
Medial malleolus					0.556
Within 2 mm	7	3.6%	5	5.1%	
Lateral malleolus					0.298
Within 2 mm	7	2.5%	7	2.5%	
Greater than 2 mm	1	0.4%	0	0.0%	
Posterior malleolus	0	0.0%	0	0.0%	1.000
Syndesmosis					0. 323
Within 2 mm	0	0.0%	2	3.2%	
Greater than 2 mm	2	1.8%	1	1.6%	

##### Call for assistance

In 2 cases (3.2%) the residents called a specialist during surgery for assistance, due to the inability to achieve a satisfactory reduction. These cases were included in an intention-to-treat analysis. It’s important to note that as the malreduction was corrected intra-operatively, these cases were not considered malreduced in our analysis.

##### Surgery time and tourniquet time

All surgeries included in our study were performed with the utilization of a tourniquet for the vast majority of the procedure, from skin to skin. While we acknowledge the reliance on tourniquet time as a surrogate measure for surgical duration, it’s important to note that this method was chosen due to inconsistencies in total surgery time documentation in the anesthesia charts. However, we recognize the inherent limitation in using tourniquet time as an exact measure, particularly considering instances where a venous tourniquet might need premature deflation. Tourniquet time was significantly longer in the resident group (87.4 min, SD 30.8) versus the specialist group (60.8 min, SD 25.5) (*p* < 0.001).

##### Discharge

The overall mean length of stay was similar between the groups (2.24 days, median 1.11, SD 3.48, *p* = 0.871).

##### Complications

The overall complications rate was 18.8% in the specialist group vs. 17.1% in the resident group (*p* = 0.690). None of the documented complications was significantly more prevalent in one group or the other (Table 4).

**Table 4:** Complications (by Rate) * *p* < 0.05

Group	Specialist *N* = 308		Residents *N* = 152		
	**N**	**%**	**N**	**%**	***p*** **-value**
Superficial Infection	16	5.2%	5	3.3%	> 0.05
Painful Hardware	14	4.5%	6	3.9%	> 0.05
Nerve Injury	10	3.2%	4	2.6%	> 0.05
Deep Infection	5	1.6%	5	3.3%	> 0.05
Deep Vein Thrombosis	5	1.6%	0	0.0%	> 0.05
Nonunion	4	1.3%	2	1.3%	> 0.05
Pneumonia	2	0.6%	1	0.7%	> 0.05
Atrial Fibrillation	1	0.3%	1	0.7%	> 0.05
CRPS	1	0.3%	1	0.7%	> 0.05
Death	0	0.0%	1	0.7%	> 0.05
None	250	81.2%	126	82.9%	0.690
Total	308	100.0%	152	100.0%	

Abbreviations: CRPS – Complex Regional Pain Syndrome

##### Reoperations

Syndesmotic screws are not routinely removed in our practice. The rate was similar between the groups of 5.7% in total (*p* = 0.836). The rate of painful hardware removal was similar between the groups (3.9%, *p* = 0.666). The rate of revision due to malreduction, nonunion, nerve injury, or infection was similar between the groups (5.2% in the specialist group vs. 7.2% in the resident group, *p* = 0.388).

### Subgroup analysis by fracture type

No significant differences were found in complications rates in the subgroups of fracture types between the groups (Supination External Rotation (SER): *p* = 0.906; Supination Adduction (SAD): *p* = 0.296; Pronation External Rotation (SER): *p* = 0.727, Pronation Abduction (PAB): *p* = 0.807). The subgroup analysis of reoperations revealed similar rates between the residents and specialists (SER: *p* = 0.166, SAD: *p* = 0.667, PER: *p* = 0.237, PAB: *p* = 0.802).

#### Subgroup analysis of the resident group by PGY

The total complication rate between PGY groups was not significantly higher in PGY 4 and 5 versus PGY 6 (29.4%, 26.3%, and 11.6% respectively, *p* = 0.170). The two PGY 3 cases (done by the same resident) had no complications. Revision rates were similar between the subgroups (*p* = 0.451).

## Discussion

Ankle fractures represent a prevalent concern within orthopaedic surgery, necessitating comprehensive training for orthopaedic surgery residents to adeptly manage these injuries.

The ‘apprenticeship model’ forms the cornerstone of medical training, particularly in surgical specialties. However, it’s crucial to acknowledge the inherent tension between surgical training and achieving optimal treatment outcomes while ensuring maximum patient safety. This study aimed to examine the impact of residents on the outcomes of ankle fracture surgeries, assessing whether the training methodology for residents has any adverse effects on treatment results.

We assessed the outcomes of 479 cases, categorizing them into two groups: surgeries conducted by unsupervised residents and those performed by specialists. Both groups exhibited comparable baseline characteristics, effectively minimizing potential confounding variables that might influence the study’s findings.

The resident group exhibited a significantly longer surgery duration compared to the specialist group, as anticipated due to the anticipated growth in surgical proficiency and efficiency with experience. Correspondingly, in Louie et al.‘s study, ankle fracture surgeries involving residents were 30 min longer than other procedures [12]. Similarly, D’souza et al.‘s meta-analysis, comprising 182 studies with 141,555 patients, indicated a mean increase of 10.2 min in surgeries involving residents [4]. Furthermore, Edelstein et al.‘s analysis of over 30,000 orthopedic surgeries through the National Surgical Quality Improvement Program (NSQIP) revealed an average extension of 17 min in surgery duration when residents participated [5].

Despite the longer duration of surgeries performed by residents compared to specialists, notably, there were no statistically significant differences in the quality of fracture reduction between the two groups. This finding stands out, as it highlights the residents’ capacity to achieve satisfactory fracture reduction comparable to specialists. Surprisingly, there is limited or no previously published data available specifically addressing the residents’ proficiency in achieving satisfactory fracture reduction. This emphasizes the novelty and importance of this study’s contribution to understanding the residents’ competency in this critical aspect of ankle fracture surgeries.

Furthermore, despite the extended duration of surgery, the complication rates remained comparable between the groups, with an overall complication rate of 18% observed in both the specialist and resident groups. Various studies have delved into the impact of resident participation in orthopedic surgeries, yielding diverse results. Louie et al., in their research on ankle fracture ORIF surgery, demonstrated a lower overall complication rate in the resident group [12]. Similarly, Edelstein et al.‘s study involving 30,628 orthopedic surgery cases revealed decreased rates of overall complications, encompassing medical complications and mortality, associated with resident participation [5]. Conversely, studies such as Gross et al. and Pugely et al. reported higher medical complication rates with resident involvement [6, 15]. Conversely, other studies indicated no influence of resident participation on outcomes, as observed in research focusing on total joint arthroplasty cases and postoperative spine infection cases [7, 8, 20, 2].

Given the wide spectrum of outcomes, from positive to negative impacts of resident participation, drawing definitive conclusions becomes challenging. Additionally, variations in defining and quantifying resident participation across studies hinder direct comparisons. Moreover, it’s essential to consider that when a senior surgeon is present during surgery, complications might be mitigated to some extent. Our belief is that evaluating the true “resident effect” on surgical outcomes necessitates isolating this variable and assessing surgeries exclusively performed by residents.

It is important to mention that, although the majority of cases within the resident group were undertaken by PGY6 residents, equivalent to the fellowship level in many systems (62%), the outcomes of PGY3-5 residents were found to be comparable to those of PGY6.

This study’s significance lies in contributing to a nuanced understanding of the implications of resident participation in surgeries, acknowledging the complexities and variations within existing literature. It modestly aims to provide a unique perspective on the role of residents in surgical outcomes, emphasizing the need for further focused investigations to delineate the true impact of resident involvement on surgical results.

However, it is essential to acknowledge several limitations inherent in this study. The retrospective nature of the study design introduces potential biases that might affect the interpretation of the results. Also, the reliance on a single-center patient cohort and a solitary residency program might restrict the generalizability of the findings to a broader population. It is also noteworthy that in numerous residency programs, the mandatory presence of a senior surgeon during surgery might impact the direct applicability of our results to such settings. Moreover, the use of tourniquet time as a surrogate for surgical duration may potentially influence certain outcomes, warranting careful consideration when interpreting the results. Furthermore, as mentioned above, the use of plain radiographs to accurately assess the quality of reduction has significant limitations. Additionally, other unaccounted factors or confounding variables related to patient characteristics or surgical variations might exist, which were not explicitly addressed in this study. Lastly, while our study encompassed all eligible surgical cases within the specified period, it primarily involved healthier patients with simple ankle fractures. This focused approach allowed for detailed analysis within this specific subset but may limit direct extrapolation to more complex cases or diverse patient populations. These limitations collectively highlight the need for cautious interpretation and emphasize the necessity for further comprehensive investigations in diverse settings to validate and refine the findings of this research.

## Conclusion

The present study illustrates that orthopaedic surgery residents, when appropriately trained, achieve comparable outcomes in ankle fracture surgery, showcasing the similar quality of reduction, complication rates, and reoperation rates as surgeries conducted by fellowship-trained attendings. These results offer valuable perspectives on the role of surgical autonomy during residency, shedding light on its significance in contemporary clinical training and surgical education.

### Electronic supplementary material

Below is the link to the electronic supplementary material.

Supplementary Material 1

Supplementary Material 2

Supplementary Material 3

Supplementary Material 4

Supplementary Material 5

Supplementary Material 6

Supplementary Material 7
